# Studies of the Morphology of Hematite Synthesized from Waste Iron Sulfate

**DOI:** 10.3390/molecules29153527

**Published:** 2024-07-26

**Authors:** Kamila Splinter, Robert Möckel, Gregor Hlawacek, Zofia Lendzion-Bieluń

**Affiliations:** 1 Department of Inorganic Chemical Technology and Environment Engineering, Faculty of Chemical Technology and Engineering, West Pomeranian University of Technology in Szczecin, Piastów Ave. 42, 71-065 Szczecin, Poland; kamila.splinter@zut.edu.pl; 2 Helmholtz-Institut Freiberg für Ressourcentechnologie (HIF), Helmholtz-Zentrum Dresden-Rossendorf (HZDR), Chemnitzer Str. 40, 09599 Freiberg, Germany; r.moeckel@hzdr.de; 3 Institute of Ion Beam Physics and Materials Research, Helmholtz-Zentrum Dresden-Rossendorf (HZDR), Bautzner Landstrasse 400, 01328 Dresden, Germany; g.hlawacek@hzdr.de

**Keywords:** iron red, morphology, microwave reaction, waste iron sulfate

## Abstract

Microwave-based reactions have gained traction in recent years due to their ability to enhance reaction rates and yield while reducing energy consumption. Also, according to the conception of ‘waste to materials’, various waste feeds are intensively sought to be tested. The experimental setup of this study involved varying pH levels, oxidation agents, and precipitation agents to optimize the synthesis process of iron red based on waste iron sulfate. The selection of oxidation and precipitation agents was found to significantly influence the pigment synthesis process. Various oxidizing agents, including hydrogen peroxide and atmospheric air, were evaluated for their effectiveness in promoting the oxidation of ferrous ions to ferric ions, essential for pigment formation. Additionally, different precipitation agents such as sodium hydroxide and ammonia solution were assessed for their ability to precipitate iron hydroxides and facilitate pigment particle formation. The characterization of synthesized pigments revealed promising results in terms of quality and color properties. Helium Ion Microscopy (HIM) analysis confirmed the formation of well-defined pigment particles with controlled morphology. X-ray diffraction (XRD) studies provided insights into the crystalline structure of the pigments, indicating the presence of characteristic iron oxide phases. By improving this technology, waste iron sulfate can be efficiently transformed into valuable iron pigments, offering a sustainable solution for waste management while meeting the growing demand for high-quality pigments.

## 1. Introduction

Iron pigments are probably one of the best-known groups of functional materials. This does not change the fact that new synthesis methods are constantly being developed to improve the properties of the materials obtained, minimize the negative impact on the environment, or use waste from other industries as raw materials for synthesis [1,2,3,4,5,6]. Waste valorization is an important aspect of economic as well as environmentally friendly production. The use of waste materials in the synthesis of materials can relieve the burden on the environment and reduce the negative impact of the chemical industry on nature. In addition, pigments synthesized based on waste raw materials have comparable or better physical and chemical properties [4]. This allows waste pigments to be used in a variety of industries where iron pigments are currently used, such as in paper coloring [7,8,9], electronics [10,11,12], paints [13,14], and cosmetics [15] (if they meet the requirements).

Obtaining pigments by precipitation is by far the simplest method for obtaining iron oxides and oxides hydroxides. The precipitation method involves adding an alkali to an aqueous salt solution containing iron ions [16,17,18]. The undoubted advantages of this method include, first of all, low production costs, short reaction time, reproducibility, homogeneity, and high conversion rate. However, the problem is the limited control of the size distribution of the obtained nanoparticles [17,19,20]. An additional problem that occurs in the precipitation method is the generation of significant amounts of waste salts, for example, Na_2_SO_4_ or NaCl.

Not only kinetic factors affect crystal growth. There are two stages in the precipitation process: nucleation—once the critical concentration is exceeded in supersaturated solutions—and slow nucleation growth by diffusion of the solute on the surface of the crystals. To obtain nanodisperse iron oxide nanoparticles with a narrow size distribution, these two stages should be separated. That is, rapid nucleation should occur first, and only then should the growth of the crystals take place. The average size of nanoparticles can be controlled by the pH and iron salt concentration [21].

In the synthesis of iron oxides, particularly hematite, various precipitation agents are employed to facilitate the nucleation and growth of nanoparticles with desired characteristics. Commonly used precipitation agents include alkalis such as sodium hydroxide (NaOH), ammonium solution (NH_3_·H_2_O), and potassium hydroxide (KOH). These alkalis play a crucial role in initiating the precipitation reaction by increasing the pH of the solution, which leads to the formation of insoluble iron hydroxide precipitates [22,23]. Among these alkalis, sodium hydroxide is frequently utilized due to its affordability, availability, and effectiveness in precipitating iron ions from aqueous solutions. Ammonium solution offers the advantage of producing precipitates under relatively mild conditions, making it suitable for controlling the morphology and size of the resulting nanoparticles. Additionally, other complexing agents or chelating agents such as ethylenediaminetetraacetic acid (EDTA) may be used in conjunction with alkalis to enhance the stability of the precursor solution and promote homogeneous nucleation [24,25,26,27].

Hematite obtained by precipitation in a neutral or low alkali pH (<9) is characterized by a spherical particle shape and wide particle distribution, from 60 to 750 nm depending on research [28,29,30]. It is shown that when using a pH higher than 9, hematite can create cubic shape particles [31]. However, cubic-shaped iron (III) oxide is obtained in high-temperature reactions (500 °C) and in long synthesis times (5–20 h) [32,33].

In contrast, the sol–gel method offers a more tailored approach to hematite synthesis [34,35]. This technique relies on the hydrolysis and condensation of metal alkoxides or salts to form a sol, which subsequently undergoes gelation and calcination to yield hematite nanoparticles [36,37]. By adjusting parameters such as precursor chemistry, solvent composition, and drying conditions, researchers can fine-tune the size, surface area, and crystallinity of the final product. The sol–gel method’s versatility and ability to produce nanoparticles with a high surface area make it particularly attractive for applications such as photocatalysis and magnetic storage [38,39,40,41].

The solvothermal method harness the unique properties of various solutions at elevated temperatures and pressures. In this method, precursor solutions containing iron salts are sealed in a pressure vessel and heated under controlled conditions, promoting the nucleation and growth of hematite crystals [42,43,44]. The hydrothermal method offers distinct advantages, including the ability to synthesize single-crystalline nanoparticles with precise control over size, shape, and crystal orientation [45,46]. However, this type of process is often results in a very long synthesis time (>10 h) [47,48,49]. In addition, its reliance on specialized equipment and operating conditions may limit its scalability and accessibility compared to other methods.

Previous studies [50,51,52] have found that synthesized pigments based on waste iron sulfate differ significantly from commercially available pigments synthesized based on pure substances. Studies have investigated the effects of the change in pressure, time of the reaction, and concentration of iron salt. Prepared pigments were characterized by a high specific surface area and low oil absorption. What is more, lengthening the time of synthesis (from 1 to 3 h) allows for increased crystallization of hematite. The increase in pressure (17–20 bars) also increases the proportion of low-size particles, resulting in a darker color of pigments. At the same time, increasing the synthesis time causes the material particles to show higher agglomeration. It is also worth mentioning that the use of microwave-based reactions allows for a significant decrease in the temperature of synthesis to 170 °C, shortening the time to a range of 1–3 h. In some samples synthesized from 14% solutions in 20 bar for 1, 2, or 3 h for the first time, interesting cubic-shaped particles were observed [50].

The current study aims to determine the differences in pigment morphology depending on the conditions under which they are synthesized. The effects of pH, the type of precipitating agent, and the oxidizing agent (Fe^2+^ → Fe^3+^) on the texture of the pigments in microwave-based reactions have been investigated.

## 2. Results and Discussion

### 2.1. XRD

**Figure 1:**
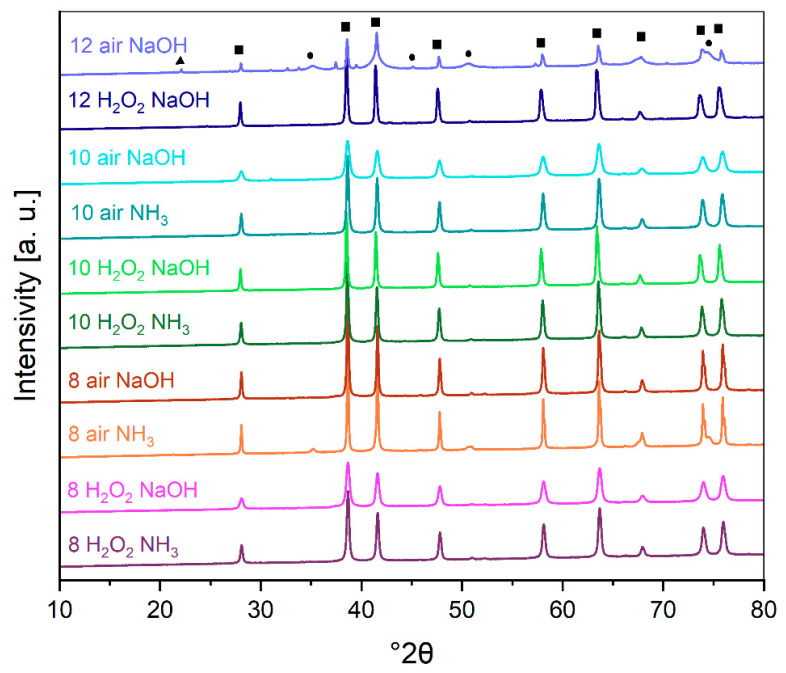
XRD pattern of laboratory pigment samples. Phases designations: square—hematite, circle—magnetite, triangle—sodium sulfate.

The phase identified in pigments is mostly fine crystalline hematite (Figure 1). In most of the samples, there are no residuals of ammonia or sodium sulfates, which is a sign of good pigment washing. In a sample named *12 air NaOH*, there are around 7% Na_2_SO_4_ residuals (Table 1), which is related to the inaccurate washing of the sample. As observed, the pH changes the consistency of suspension, and the same methodology of washing is not enough to get rid of residuals of soluble salts. Goethite was identified in some samples, namely *10 H_2_O_2_ NaOH*, *10 H_2_O_2_ NH_3_*, and *12 H_2_O_2_ NaOH*. However, due to low content (below 3 wt%), it is hardly visible in Figure 1.

The content of only well-crystalline hematite characterizes pigments oxidized with hydrogen peroxide. Air-oxidized pigments, for example, *8 air NH_3_*, regardless of the length of the oxidation carried out, also show in their composition the presence of magnetite (<20 wt%). In addition, the pigments for the precipitation of which NaOH was used are characterized by a very wide range of crystallite sizes (30–350 nm). Pigments for the precipitation of which ammonia water was used are characterized by a narrow range of crystallite sizes (30–90 nm) independent of pH and the oxidant used.

### 2.2. FTIR

**Figure 2:**
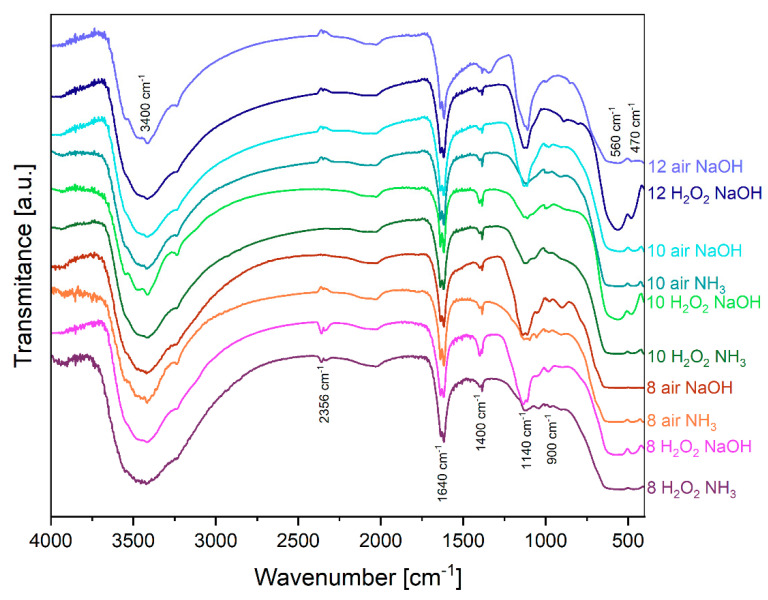
FTIR spectra for obtained materials.

At positions 470 cm^−1^ and 560 cm^−1^ bands typical for iron oxides can be observed. Fe-O bond at these wavelengths, however, cannot distinguish between hematite (α-Fe_2_O_3_), goethite (α-FeOOH), or magnetite (Fe_3_O_4_) [3,51,52,53,54,55,56,57] (Figure 2). Other band characteristics related to the Fe-O functional group for maghemite (γ-Fe_2_O_3_), goethite, and hematite, respectively, appear in small bumps in 900 cm^−1^ and 1400 cm^−1^ [58], and again, it is hard to differentiate for which compound the band is characteristic. It seems that the majority of the samples are hematite due to the low transmittance of hematite bands, which is in correlation to the XRD results.

The broad-stretching band at 3400 cm^−1^ is concerned with an H-O-H stretching band of water molecules within the crystal structure of hematite. This broad band is due to the hydrogen bonding between hydrogen and oxygen of different water molecules [59]. The other band from H-O-H appears at 1640 cm^−1^, and it is characteristic of bending vibration [3,53,57,60].

The band around 2356 cm^−1^ is from incidentally absorbed CO_2_ in the surface of the tablet during measurement. The band at position 1140 cm^−1^ can be connected with the S-O band. The highest intensity of this band occurred with samples precipitated by sodium hydroxide and can be connected with residuals of sodium sulfate in samples that are less soluble in water than ammonia sulfate [61,62]. However, this band appears in every sample, due to the existence of sulfates in the reaction environment as a precursor (FeSO_4_).

### 2.3. BET

**Figure 3:**
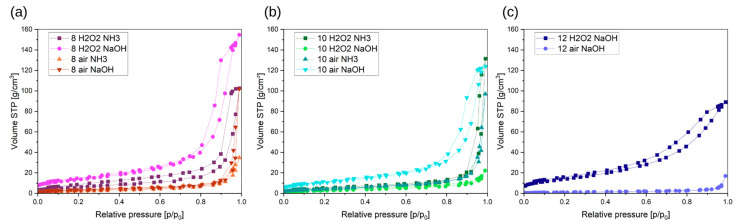
Results obtained from BET analysis. Nitrogen adsorption isotherm of pigments synthesized in pH ranges (**a**) 8, (**b**) 10, and (**c**) 12.

**Figure 4:**
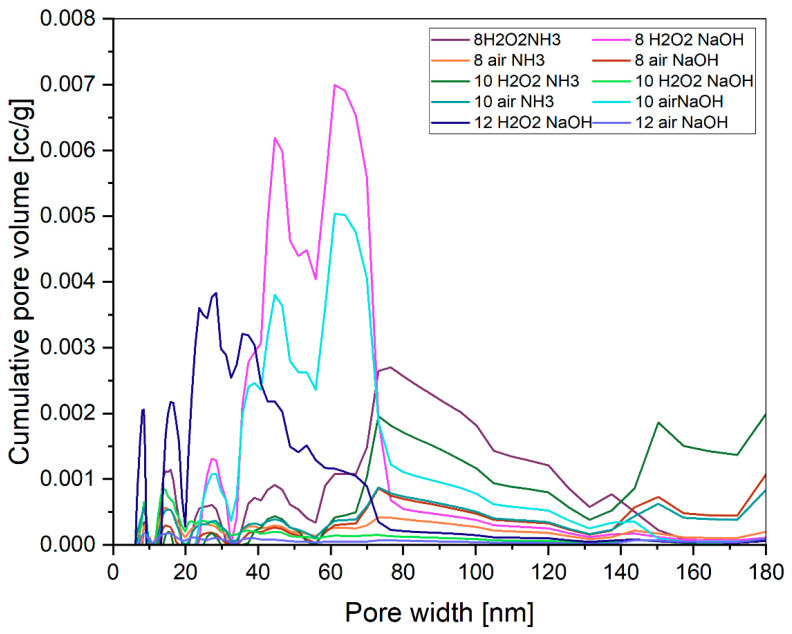
DFT diagrams for obtained pigments.

Obtained pigments have an isotherm shape corresponding to types IV and V according to the IUPAC classification [63,64] (Figure 3). It is a typical characteristic of mesoporous materials. The hysteresis shape corresponds to the H3 type, i.e., slit-shaped pores. There is no major difference in the isotherm shape between samples. However, according to the nomenclature proposed by IUPAC [63], several samples can be referred to as macroporous materials. DFT shows that some of the materials are actually mesoporous, so they have the largest proportion of pores in the 2–50 nm range (Figure 4). In contrast, some samples show the highest proportion of pores above 50 nm, making them macroporous. It should be noted, however, that the analyzed materials have a small total pore volume of <0.2 cm^3^/g.

**Figure 5:**
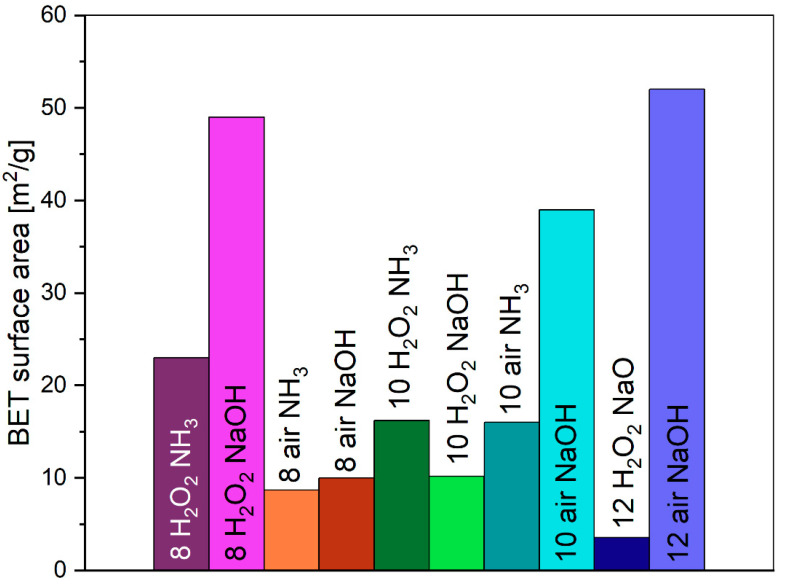
BET-specific surface area of laboratory pigments.

Synthesized pigments have up to five times greater specific surface area than commercial pigments (values up to 10 m^2^/g) [65]. Pigments synthesized by microwave-based reaction have a specific surface area in the range of 4–52 m^2^/g (Figure 5). Oxidation agents do not affect the specific surface area much; however, pigments precipitated with sodium hydroxide are characterized mostly by higher specific surface area values.

### 2.4. HIM

**Figure 6:**
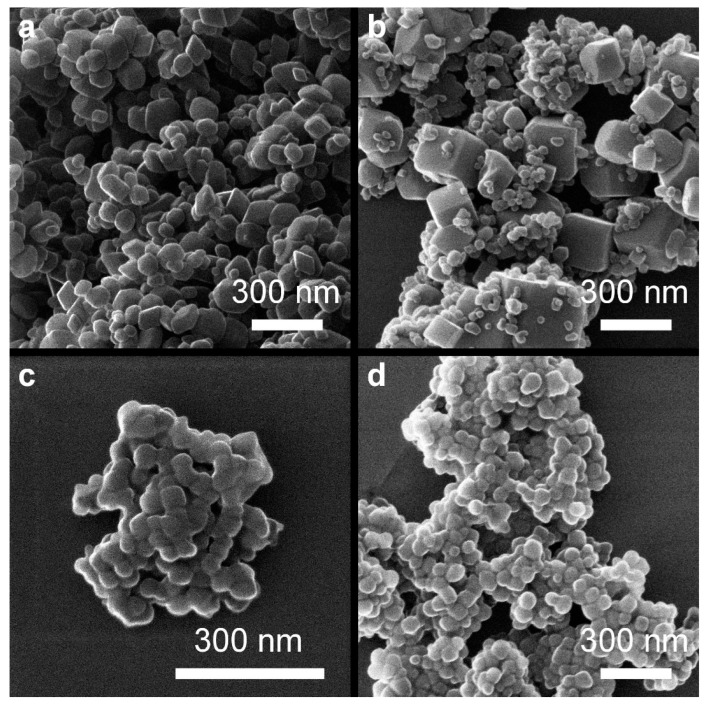
Overview of samples: (**a**) *8 air NaOH*, (**b**) *8 air NH_3_*, (**c**) *8 H_2_O_2_ NaOH*, and (**d**) *8 H_2_O_2_ NH_3_*.

**Figure 7:**
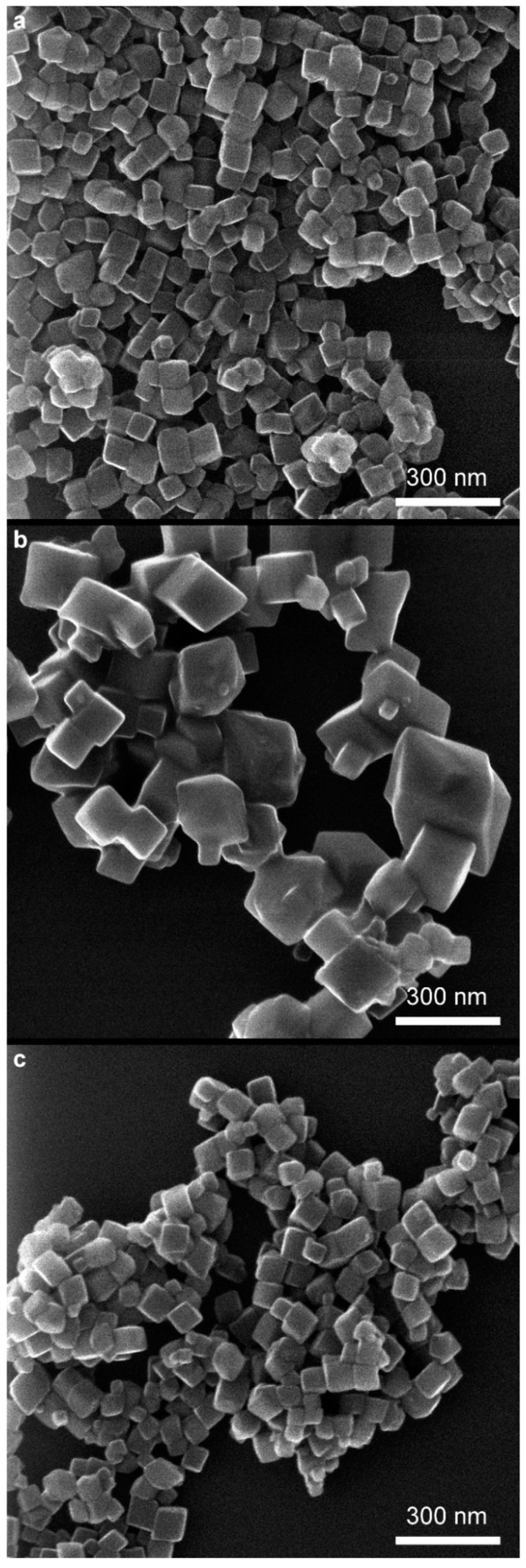
Overview of samples: (**a**) *10 air NH_3_*, (**b**) *10 H_2_O_2_ NaOH*, and (**c**) *10 H_2_O_2_ NH_3_*.

**Figure 8:**
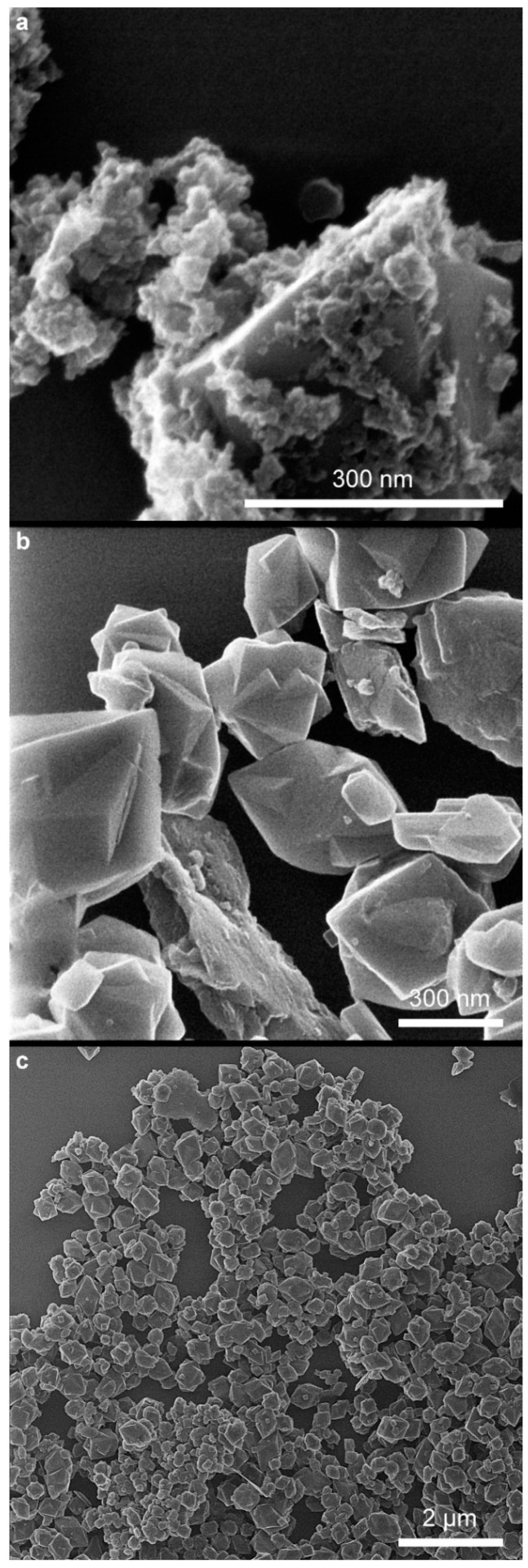
Overview of samples: (**a**) *12 air NaOH*, (**b**) *12 H_2_O_2_ NaOH*, and (**c**) *12 H_2_O_2_ NaOH*.

**Figure 9:**
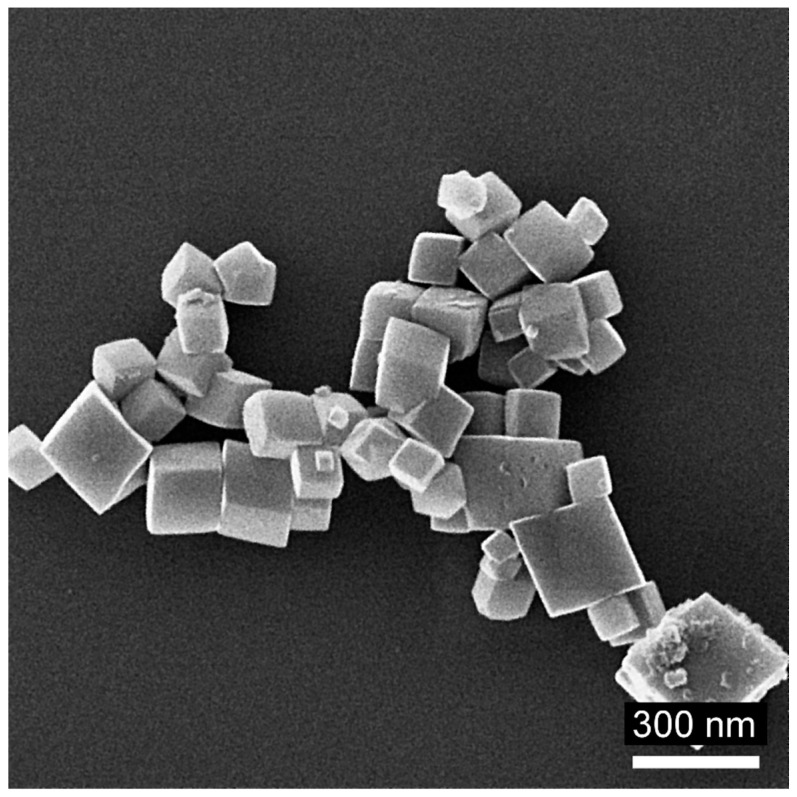
HIM image of reference material from previous research—14%, 20 bar, 3 h [50].

From analyzing the HIM images (Figure 6, Figure 7 and Figure 8), it can be concluded that the samples synthesized at pH = 8 have an irregular particle shape, similar to cubes. In the case of sample *8 air NaOH*, we observe an interesting structure—large hematite particles on which finely crystalline magnetite has been deposited, as confirmed by XRD results. Samples synthesized at pH = 10 are characterized by visible cubic particle shapes with small particle sizes, confirming the XRD and DLS results. The same cubic particle texture has been observed in previous studies [50] (Figure 9). For samples synthesized at pH = 12, we see a rather irregular particle texture. In the case of sample *12 H_2_O_2_ NaOH* (Figure 8c), it can be concluded at first that the sample is very homogenous with larger particles than in other syntheses. However, looking into a deeper texture (Figure 8b) it is seen that the structure is for sure not as homogenous as at first sight—as seen in the rhombic shape of particles with many defects. Most of the samples are homogenous in the size of particles.

### 2.5. DLS

**Figure 10:**
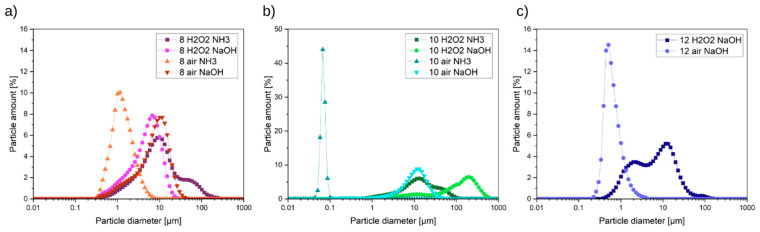
Pigment size distribution analysis by dynamic light scattering of pigments synthesized in a pH range of (**a**) 8, (**b**) 10, and (**c**) 12.

Pigments are characterized by different particle size distributions (Figure 10). Many samples are characterized by a narrow size distribution of particles in a range of 0.1–1.5 µm.

Interesting particle size distribution is shown by samples *8 H_2_O_2_ NaOH* and 8 air NaOH. Both samples are characterized by the same shape of particle size distribution only shifting toward higher values in the case of the *8 air NaOH* sample.

However, the DLS results are not aligned with the XRD and HIM results. For example, in the case of *8 air NH_3_* samples when analyzing their DLS results, it can be concluded that the sample is homogenous. However, in reality, as seen in XRD and in the HIM image, that sample consists of two phases with different sizes of particles.

### 2.6. Oil Number

**Figure 11:**
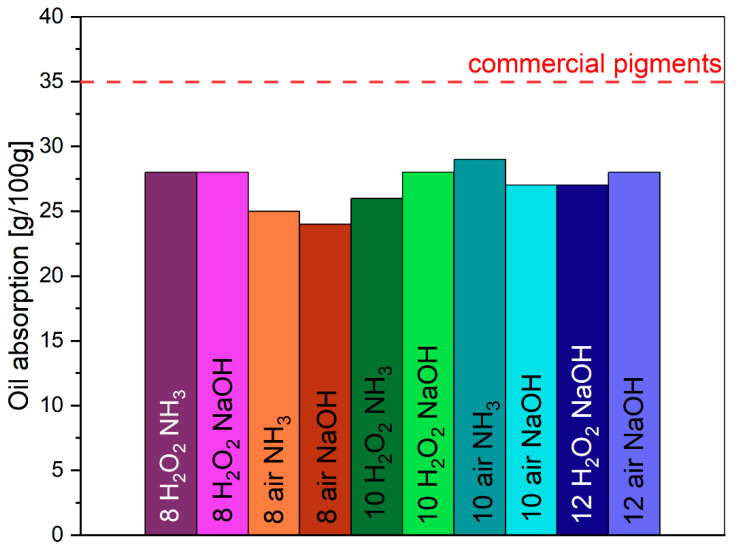
Oil absorption for obtained pigments.

The oil absorption of synthesized pigments is in the maximum value of 35 g/100 g [65]. Microwave-based pigments in this study have much a lower oil absorption rate in the range of 24–28 g/100 g (Figure 11). It is connected with good crystallinity of materials and a low specific surface area.

Due to the planned use of the resulting pigments in paints and for coloring building materials, a low oil number is one of the critical indicators. The low index makes it possible to use the color pigment in the smallest possible amount, and the viscosity of the system will be controlled by other, often cheaper nanofillers such as chalk or silica.

### 2.7. Color Analysis

According to the mathematical CIELAB color theory, Table 2 presents values *L*, *a*, *b*, and *ΔE*, color codes in hexadecimal, and the color interpretation of HEX codes [66].

The luminance of pigments is on an average level for dark-medium red pigments. However, luminance for pigments precipitated with ammonia water or oxidized by air is lower than in pigments precipitated by sodium hydroxide or oxidized by hydrogen peroxide. Values a and b do not change significantly according to precipitation and oxidation agents, nor according to pH.

However, pigments precipitated in pH = 8 have the best redness parameters—value a is the highest in the samples batch. The higher redness values for these pigments can be connected with narrow particle size distribution shifted into higher values (with the highest peak around 1 µm). This phenomenon can be caused by the agglomeration of particles and changes in the optical properties of pigment.

## 3. Materials and Methods

### 3.1. Materials

Waste FeSO_4_ from Grupa Azoty Zakłady Chemiczne POLICE was used. The waste was purified using the recrystallization method and the obtained purified FeSO_4_ was used as the feed material for iron pigment synthesis. A detailed description of the purification method is available in the publication [65,67] and patent [68].

Reagent purity substances were also used: 10 wt% sulfuric acid (Chempur, Piekary Śląskie, Poland), 25 wt% ammonia water (Chempur, Poland), 3.9 wt% sodium hydroxide (Chempur, Poland), and 30 wt% hydrogen peroxide (Chempur, Poland).

### 3.2. Pigment Preparation

The effects of the pH (8, 10, or 12), precipitation (25 wt% ammonia solution or 1M sodium hydroxide), and oxidation agents (30 wt% hydrogen peroxide or air) were investigated. For this purpose, a salt solution with a concentration of 140 g/L of purified FeSO_4_ was prepared. First, the assumed amounts of purified FeSO_4_ [65,68] were stirred at room temperature until they formed a transparent light-green solution in distilled water.

Hydrogen peroxide (10 g) or air was used to oxidize the solution. Oxidation by hydrogen peroxide was conducted by mixing an assumed amount of salt solution with H_2_O_2_ and stirring for 10 min until the solution turned yellow-brown and foam from decomposed hydrogen peroxide stopped appearing. A total of 10g of H_2_O_2_ was an excess of the assumed salt content. In case of oxidation by air, the mixture was stirred for 30 min and aerated with atmospheric air until the solution turned yellow-brown. Then, to precipitate the iron(III) hydroxide, ammonia or sodium hydroxide solutions were added. Depending on the samples, a precipitation agent was added until the pH was 8, 10, or 12. However, using ammonia water, the maximum pH value achieved with precipitation was 11.56. For this reason, the article does not include samples precipitated with ammonia water at pH = 12.

The suspension was then transferred into a Teflon container at a volume of 70 mL, and placed in a microwave reactor (Ertec Magnum II). The reaction time was 1 h, the temperature of the reaction was about 160–165 °C, and the pressure was set at 17–20 bar based on previous research [65,67]. Due to the use of a microwave reactor, minimum and maximum pressure settings are required. The difference of 3 bar is the minimum difference of pressure to ensure the proper operation of the reactor. At the end of the stage, the obtained suspensions were washed with water and dried at 105 °C for 4 h.

In order to distinguish between samples, names were introduced according to the following example: *8 H_2_O_2_ NaOH*, meaning that the sample was oxidated by hydrogen peroxide and precipitation was made with sodium hydroxide until pH = 8. Other used designations are as follows: air—oxidated by air, NH_3_—precipitated by ammonia water, and 10 or 12 are, respectively, designations of the end pH of precipitation. A detailed description of each sample is available in Table 3.

### 3.3. Methods

XRD patterns were collected with an X-ray diffractometer (Empyrean, Malvern Panalytical, Almelo, The Netherlands) equipped with a Co-Kα radiation source (iron filter, λ = 0.1789 nm, 35 kV, 35 mA) and a PIXcel3D-Medipix 1 × 1 area detector. Scans were taken at room temperature in a scattering 2θ range of 5–80° with a step interval of about 0.0131°. The irradiated area was kept constant at 15 × 12 mm^2^ by using an automated divergence slit and a 15 mm beam mask. The phase composition was determined using Panalytical X’Pert HighScore Plus v3.0 software with the ICDD PDF4+ database. Quantifications were carried out utilizing the so-called Rietveld method through the BGMN/Profex software package v. 5.0 [69].

Fourier transform infrared spectroscopy (FTIR) spectra of the sample were measured in a range of 4000–400 cm^−1^ on a Nicolet 380 spectrometer (Thermo Fisher Scientific, Waltham, MA, USA). The sample was mixed with KBr at a ratio of 1:100 and then compressed into tablets.

The specific surface area of the pigments was determined by the Brunauer–Emmett–Teller (BET) method using a Quadrasorb Evo Quantachrome Instruments nitrogen adsorption apparatus. A sample was degassed at 100 °C under a high vacuum for 16 h. Adsorption and desorption isotherms, as well as pore size distributions in the density functional theory (DFT), were determined with QuadraWin v. 7.1.

Dynamic light scattering measurements (DLS) were carried out on a Horiba LA 950 Laser Diffraction Particle Size Analyzer. The sample was dispersed in 50 mL of 0.1% sodium pyrophosphate solution. Then, the solution was sonicated in an ultrasonic cleaner for 2 min. The prepared dispersion was loaded into the analyzer. In the measurements, a reflectance coefficient of 2.90 was used, and sonication was turned on.

The surface morphology of the samples was investigated with the help of a Helium Ion Microscope (HIM) [70] (Carl Zeiss NanoFAB). The samples were dispersed in isopropyl alcohol and placed on silicon wafers to allow the solvent to evaporate. Images were recorded using 25 keV He beams and a current of 250 fA, 10 µs pixel dwell time, and 8 lines averaging. For selected images, charge compensation was necessary [71,72]. In these cases, averaging over 128 lines with a pixel dwell time of 1 µs was used. A flood gun was used after each scan line for 300 µs with an electron energy of 480 eV and a flood time of 300 ms.

Oil absorption was determined according to the PN-EN ISO 787-5:1999 standard [73].

Color analysis was conducted with a hand spectrophotometer (Konica Minolta, CM-700d, Tokyo, Japan). The spectrometer was calibrated on a white standard and three measurements for each sample were performed. The CIELAB scale was used to measure pigment color. This scale is based on the theory of opposing colors, assuming that receptors in the human eye perceive colors as pairs of opposites. Designed in this way, the scale allows a given color to be placed in three-dimensional space, using three values—*L*, *a*, and *b*—to describe each color [50,74].

## 4. Conclusions

The effects of pH, the type of precipitating agent, and the oxidizing agent on the texture of the pigments have been investigated.

In the investigation, it was observed that hydrogen peroxide (H_2_O_2_) serves as a superior oxidizing agent compared to air. Notably, H_2_O_2_ facilitates more precise control over the oxidation process. This enhanced control is pivotal for achieving desired pigment characteristics, such as texture and color consistency.

Furthermore, experiments conducted at a pH level of 10 revealed the most homogeneous particle size distribution among the samples. This finding suggests that pH plays a crucial role in determining the uniformity of pigment particles, with a pH of 10 being optimal for achieving homogeneity. Potential agglomeration can be avoided by using surface modifications.

Moreover, at a pH of 8, the pigments exhibited the most favorable color parameters. This indicates that pH manipulation can significantly impact the color properties of the synthesized pigments, with a pH of 8 yielding the most desirable color characteristics.

In terms of surface area, sodium hydroxide (NaOH) was found to produce pigments with the largest Brunauer–Emmett–Teller (BET) surface area. This suggests that the choice of precipitating agent can directly influence the surface properties of the synthesized pigments, potentially affecting their application properties.

Finally, the presence of magnetite in the samples was observed to darken the color of the pigments. This suggests that the composition of the precipitated pigments, particularly with the inclusion of magnetite, can impact their final coloration.

In summary, the investigation highlights the intricate interplay between pH, the type of precipitating and oxidizing agents, and pigment morphology. While pH primarily influences particle homogeneity and color properties, the choice of oxidizing and precipitating agents can significantly affect texture, color, and surface properties. Moreover, the superior performance of hydrogen peroxide as an oxidizing agent underscores the importance of precise control in pigment synthesis processes. Specifically, the research demonstrated the conversion of waste iron sulfate into hematite, highlighting an innovative approach to waste management. Transforming waste materials into valuable products contributes to environmental sustainability and resource efficiency. This valorization process not only reduces waste but also offers a practical solution for utilizing industrial by-products. These findings provide valuable insights for optimizing the synthesis of pigments for various industrial applications and pave the way for research on pigment modification. Also, these findings underscore the importance of rethinking waste as a resource.

## 5. Patents

Splinter, K., Lendzion-Bieluń, Z., and Wojciechowska, A. (2021) Method of producing iron pigments. Granted patent: Pat.245453 at 20.05.2024.

## Figures and Tables

**Table 1:** Summary of content and crystallite size for each phase identified in the samples.

Sample ID	Phase	Content [wt%]	Crystallite Size [nm]
*8 H_2_O_2_ NH_3_*	Hematite	100	50
*8 H_2_O_2_ NaOH*	Hematite	100	30
*8 air NH_3_*	Hematite	85	110
	Magnetite	15	29
*8 air NaOH*	Hematite	100	62
*10 H_2_O_2_ NH_3_*	Goethite	2	11
	Hematite	98	63
*10 H_2_O_2_ NaOH*	Goethite	2	22
	Hematite	98	73
*10 air NH_3_*	Hematite	98	55
	Magnetite	2	68
*10 air NaOH*	Hematite	100	25
*12 H_2_O_2_ NaOH*	Goethite	4	40
	Hematite	96	60
*12 air NaOH*	Na_2_SO_4_	7	-
	Hematite	32	144
	Magnetite	61	9

**Table 2:** Summary of *L*, *a*, *b*, and *ΔE* values for samples. The color codes are given in hexadecimal (HEX).

Sample	*L*	*a*	*b*	*ΔE*	Color Codes (HEX)	Color
*8 H_2_O_2_ NH_3_*	35.4	49.5	18.2	82.8	9A243A	
*8 H_2_O_2_ NaOH*	36.0	47.2	17.0	80.8	992A3D	
*8 air NH_3_*	19.9	30.7	18.5	87.1	5A1817	
*8 air NaOH*	29.8	42.2	15.7	82.8	802231	
*10 H_2_O_2_ NH_3_*	35.5	25.6	11.5	69.6	7C4243	
*10 H_2_O_2_ NaOH*	37.0	32.5	11.5	71.2	893F46	
*10 air NH_3_*	22.7	15.8	11.5	79.0	4F2D26	
*10 air NaOH*	31.2	27.8	11.6	74.4	733639	
*12 H_2_O_2_ NaOH*	29.6	37.2	11.3	79.8	7A2836	
*12 air NaOH*	19.8	40.8	15.5	90.7	64021C	

**Table 3:** Table with the names of all samples with the corresponding conditions.

Sample ID	Oxidation Agent Used	Precipitation Agent Used	pH
*8 H_2_O_2_ NH_3_*	Hydrogen peroxide	Ammonia solution	8
*8 H_2_O_2_ NaOH*	Hydrogen peroxide	Sodium hydroxide	8
*8 air NH_3_*	Air	Ammonia solution	8
*8 air NaOH*	Air	Sodium hydroxide	8
*10 H_2_O_2_ NH_3_*	Hydrogen peroxide	Ammonia solution	10
*10 H_2_O_2_ NaOH*	Hydrogen peroxide	Sodium hydroxide	10
*10 air NH_3_*	Air	Ammonia solution	10
*10 air NaOH*	Air	Sodium hydroxide	10
*12 H_2_O_2_ NaOH*	Hydrogen peroxide	Sodium hydroxide	12
*12 air NaOH*	Air	Sodium hydroxide	12

## Data Availability

The data presented in this study are available on request from the corresponding author.
